# Structural comparison of somatically related PG9 and PG16 in complex with their epitope reveals differences in glycan recognition

**DOI:** 10.1186/1742-4690-9-S2-P74

**Published:** 2012-09-13

**Authors:** M Pancera, JS McLellan, S Shahzad-ul-Hussan, N Doria-Rose, B Zhang, Y Yang, DR Burton, WC Koff, CA Bewley, PD Kwong

**Affiliations:** 1 NIH/NIAID/VRC, Bethesda, MD, USA

## Background

The somatically related antibodies, PG9 and PG16, neutralize 70-80% of HIV-1 isolates and bind a glycosylated epitope in the V1/V2 domain of HIV-1 gp120. Mutations in V1/V2, and sometimes V3 depending on the HIV-1 strain, affect neutralization and a glycan on Asn160 is required for neutralization. Both antibodies also preferentially bind the native trimer over monomeric gp120, especially PG16. The structure of PG9 in complex with its epitope, a scaffolded V1/V2 from HIV-1 strain ZM109, was recently solved and showed that PG9 targets a site of vulnerability comprising 2 glycans and a β-strand.

## Methods

To understand the differences in binding properties from these two somatically related antibodies, we first assessed their binding to monomeric gp120 and scaffolded V1/V2 proteins with different glycan types (oligomannose, hybrid, and complex). In order for PG16 to bind the scaffolded V1/V2, the protein had to be expressed in mammalian cells in the presence of swainsonine, which inhibits glycan maturation past the hybrid state. A stable complex could be obtained between PG16 and a scaffolded V1/V2 domain from ZM109, and this complex was crystallized.

## Results

Although the structure of PG16 bound to scaffolded V1/V2 resembled that of PG9, some differences were seen: 1) PG16 binding to the β-strand is weaker than PG9 with fewer charged interactions, 2) PG16 interacts with a hybrid glycan at position N173. The difference in binding recognition of PG9 and PG16 to monomeric gp120 depends on the type of glycans present. PG16 binds the protein portion of V1/V2 weaker than PG9 and this might explain the higher affinity of PG9 for the monomer. PG16 has evolved a second glycan site to compensate for weaker peptide interaction.

## Conclusion

The results show the importance of polyclonal response in infected individual to combat HIV-1, and in this case, to differential glycosylation.

